# Comparative Effects of Probiotics and Paraprobiotics Derived from *Lactiplantibacillus plantarum*, *Latilactobacillus sakei*, and *Limosilactobacillus reuteri* in a DSS-Induced Ulcerative Colitis Mouse Model

**DOI:** 10.4014/jmb.2411.11045

**Published:** 2025-02-25

**Authors:** Yun Young Kang, Hyo Jeong Song, So Young Park, Dong Nyoung Oh, Ga Yeong Kim, Na Yeong Been, Da Yeong Kim, Eun Ji Lee, Bo-Hye Nam, Jong-Min Lee

**Affiliations:** 1 Department of Biotechnology, Pukyong National University, Busan 48513, Republic of Korea; 2 Aquaculture Research Division, National Institute of Fisheries Science, Busan 46083, Republic of Korea

**Keywords:** Paraprobiotics, probiotics, anti-inflammation, tight junction, ulcerative colitis

## Abstract

Live biotherapeutic products, represented by probiotics with disease-mitigating or therapeutic effects, face significant limitations in achieving stable colonization in the gut through oral administration. However, paraprobiotics, which consist of dead or inactivated microbial cells derived from probiotics, can provide comparable health benefits while overcoming the limitations associated with live biotherapeutic products. Therefore, the purpose of this study was to quantitatively compare and analyze the effects of probiotics, which are gaining attention as treatments for inflammatory bowel diseases, and their paraprobiotic counterparts on the alleviation of ulcerative colitis. In *in vitro* evaluations revealed that the paraprobiotics derived from *Lactiplantibacillus plantarum* MGEL20154, *Latilactobacillus sakei* MGEL23040, and *Limosilactobacillus reuteri* MGEL21001 exhibited equal or significantly enhanced activities in terms of antioxidant properties, anti-inflammatory effects, and barrier integrity enhancement compared to their probiotic counterparts. Furthermore, consistent with *in vitro* findings, both probiotics and paraprobiotics effectively improved histological scores and reduced myeloperoxidase levels in a DSS-induced ulcerative colitis mouse model. Notably, paraprobiotics derived from *L. plantarum* MGEL20154 and *L. reuteri* MGEL21001 demonstrated significantly enhanced efficacy in restoring tight junctions, promoting mucin secretion, and reducing inflammation in colonic lesion tissues compared to their probiotic forms. Our results suggest that these paraprobiotics may serve as more suitable agents for alleviating and treating ulcerative colitis, addressing limitations associated with probiotics, such as low survival rates, instability, antibiotic susceptibility, and the potential induction of excessive inflammatory responses.

## Introduction

Probiotics are live microorganisms that confer health benefits when consumed in appropriate amounts [1]. Typically, lactic acid bacteria, bifidobacteria, and some eukaryotic microorganisms, such as yeast, are considered representative probiotics. Numerous clinical studies have confirmed the human health of probiotics, including improvement of intestinal microbial balance, immune system regulation, and enhancement of digestion and bowel function [2-5]. Therefore, probiotic consumption has become widespread as a functional food that helps maintain health and prevent disease. Moreover, probiotics are increasingly being explored as a foundation for the development of microbiome drugs, representing a novel approach to disease treatment. Microbiome drugs utilize specific live biotherapeutic products (LBPs) to treat or alleviate diseases caused by an imbalance in the intestinal microbial flora [6]. In this regard, probiotics are being used as LBPs, acting as the key active ingredients in microbiome drugs. Notable examples of microbiome drugs include “Rebyota,” approved for rectal injection, and “Vowst,” for oral administration, both of which have received approval from the U.S. Food and Drug Administration (FDA) for the treatment of *Clostridium difficile* infection. The application of probiotics has thus expanded from functional foods to active ingredients in LBPs. This shows that the benefits of probiotics extend beyond their health promotion effects, and are promising candidates for innovative therapeutic approaches.

Probiotics offer numerous benefits in maintaining physiological homeostasis and in the treatment and alleviation of diseases; however, significant limitations affect their oral administration and their stable colonization in the intestines, which are essential for their effectiveness [7]. First, survival and competition in the gastrointestinal tract present challenges. The harsh conditions of the gastrointestinal tract, such as low pH, bile salts, and the presence of digestive enzymes, pose serious threats to the survival of probiotic strains, significantly reducing their viability before they reach their intended site of action in the intestines. Moreover, upon reaching the intestines. Furthermore, once in the intestines, probiotics must compete with resident gut microbes for nutrients, attachment sites, and other resources. This competition limits their ability to colonize and establish a stable presence in the gut, potentially reducing their effectiveness. Second, susceptibility to antibiotics poses another challenge. Antibiotic susceptibility is essential for the safety of probiotics, it also makes them vulnerable. Probiotics are sensitive to a variety of antimicrobial agents, including antibiotics, present in the intestines due to medical treatment or dietary intake, which can drastically reduce their stability and colonization in the gut. Third, maintaining an adequate number of viable cells is essential for probiotics to be effective over time. To ensure functionality, a sufficient quantity of viable bacteria must be maintained for a certain period. Most products contain dried live microorganisms in a powdered form; however, their shelf-life for maintaining viability is often limited, making it challenging to guarantee an appropriate number of viable bacteria [8]. This can lead to discrepancies between the labeled probiotic content and the actual viability in commercial products, affecting product safety and reliability. Finally, the risk of illegal leakage of functional strains is a significant concern. Since live cells are directly included in the product, companies face the risk of patented strains being leaked, making these strains available to competitors or other third parties. Such leaks can undermine the company’s exclusive market position and competitiveness, potentially leading to legal disputes that consume time and resources, ultimately impacting the originality and credibility of the product and brand. In summary, when probiotics enter the human body, their stability is challenged by various factors, including the harsh gastrointestinal environment, competition with resident intestinal microorganisms, susceptibility to antimicrobial agents, maintenance of viable bacterial counts, and security concerns.

Paraprobiotics, which are killed or inactivated microbial cells or their components that are not live probiotic strains, offer similar health benefits to probiotics while overcoming their limitations [9]. Paraprobiotics can provide physiological benefits akin to probiotics through the direct ingestion of cellular components, thereby bypassing the need for survival and competition in the gastrointestinal tract and avoiding antibiotic sensitivity. They contribute to immune regulation, suppression of pathogenic bacteria, and enhancement of intestinal health [10]. From a safety perspective, the overproduction of organic acids, harmful metabolic activities, and excessive immune stimulation by live bacteria can induce inflammation in the intestinal barrier [11]. If the intestinal barrier is compromised, live probiotics may cross into the bloodstream and spread to other organs, potentially causing unexpected side effects. However, as paraprobiotics are metabolically inactive, they can be used safely in patients with weakened immune systems or those in high-risk groups. Paraprobiotics are also more resistant to environmental stresses, such as heat, pH, and oxidation, compared to live bacteria. They demonstrate high stability during manufacturing, storage, and transportation, so eliminating the need for refrigeration, enabling easy storage at room temperature, and extending shelf life. Additionally, paraprobiotics have a low risk of spoilage and contamination, making it easier to maintain product quality. They can also be developed into various dosage forms, such as powders, capsules, and tablets, providing convenience for consumers. Moreover, products containing dead cells eliminate the risk of external leakage of live bacteria. In this way, paraprobiotics are gaining attention as an alternative to probiotics due to their functionality, safety, stability, convenience, and prevention of technology leakage.

This study aimed to conduct a comparative analysis of the advantages of paraprobiotics in the form of dead cells against living bacteria in certain functional aspects. The target probiotic strains included *L. plantarum* MGEL20154, *L. sakei* MGEL23040, and *L. reuteri* MGEL21001, which represent nomadic, free-living, and host-adapted strains, each with established functionality from previous laboratory studies [7, 12, 13]. Accordingly, both probiotics and paraprobiotics from these three strains were quantitatively assessed for their antioxidant activity, inflammation-regulating effects, and intestinal integrity-restoring capabilities in *in vitro* assessments. Furthermore, the efficacy of these strains in disease alleviation was comparatively validated *in vivo* using a DSS-induced ulcerative colitis model in mice. Our findings suggest that, at minimum, the three tested strains of paraprobiotics hold promise as alternatives to probiotics, potentially overcoming probiotic limitations and providing health benefits and mitigating colitis.

## Materials and Methods

### Bacterial Strains and Culture Conditions

The bacterial strains used in this study included *L. plantarum* MGEL20154, *L. sakei* MGEL23040, *L. reuteri* MGEL21001, and *Staphylococcus aureus* KCTC 1928. Each lactic acid bacterium and the indicator strain were cultured at 37°C in a 5% CO_2_ atmosphere in De Man, Rogosa, and Sharpe (MRS; Difco, Becton, Dickinson and Co., USA) medium and brain heart infusion (BHI; Difco) medium, respectively.

### Cell Culture and Cytotoxicity

Human epithelial Caco-2 cells (KCLB 30037) were maintained at 37°C in a humidified 5% CO_2_ atmosphere in minimal essential medium (MEM; Sigma-Aldrich, USA) supplemented with 10% heat-inactivated fetal bovine serum (FBS) and 1% penicillin/streptomycin. Mouse macrophage RAW 264.7 cells (KCLB 40071) were cultured in Dulbecco’s modified Eagle’s medium (DMEM; Sigma-Aldrich), supplemented with 10% FBS and 1% L-glutamine, and incubated at 37°C in 5% CO_2_. Cytotoxicity was investigated using the Cell Proliferation Assay Kit I, which employs a colorimetric assay based on 2,5-diphenyl-2H-tetrazolium bromide (MTT) (Sigma-Aldrich) in accordance with the manufacturer’s protocol.

### Bacterial Identification

The 16S rRNA gene sequence was amplified using primers 27F (5'-TACCAGGGTATCTAATCC-3') and 1492R (5'-GGTTACCTTGTTACGACTT-3'), and sequenced using BigDye Terminator v3.1 Cycle Sequencing Kits (Applied Biosystems, USA) [14]. Phylogenetic analysis based on 16S rRNA gene sequences (>1,400 base pairs (bp)) was performed using MEGA 7 software (RRID:SCR_000667). Phylogenetic distances were calculated using the neighbor-joining, maximum-likelihood, and maximum-parsimony methods. Each resultant tree was evaluated by bootstrap analysis with 1,000 replications. The 16S rRNA gene sequence similarities between closely related species were compared and calculated using the GenBank database (http://www.ncbi.nlm.nih.gov; RRID:SCR_006472). Morphological examination was conducted using scanning electron microscopy (SEM; JSM-6490LV, Jeol, Japan) with Gatan mono CL3+ (Gatan, USA) at a voltage of 10 kV on cells grown for 12 h on MRS agar plates at 37°C in a 5% CO_2_ atmosphere.

### Probiotics and Paraprobiotic Preparation

Cultures of lactic acid bacteria incubated in MRS broth for 18 h were centrifuged at 11,000 ×*g* for 10 min and washed twice with pH 6 phosphate-buffered saline (PBS). Viable cells were counted using the serial dilution method and calculated as colony-forming units (CFU) [15]. The cell concentration was adjusted to 10^10^ CFU ml^−1^ in PBS, and these viable cells were used as probiotics. A portion of the cell suspension adjusted to 10^10^ CFU ml^−1^ was autoclaved at 121°C for 15 min for the preparation of paraprobiotics. The heat-killed probiotics were further inactivated and homogenized by sonication in an ultrasonic bath (Power Sonic 520; Hwashin Tech., Republic of Korea) for 10 min. The absence of viable cells was confirmed after 24 h of incubation by spreading the paraprobiotic samples on MRS agar plates. Paraprobiotic samples were freshly prepared for each experiment, with some samples freeze-dried and stored at 4°C for future use.

### Stability Test according to Temperature

Prototype probiotic products containing a viable cell count of 10^10^ CFU were prepared to evaluate the stability of probiotics at various temperatures. The prototypes were formulated by mixing 20% skim milk powder, 50%fructooligosaccharides, 10% xylitol, 0.1% vitamin C, and 20% lyophilized probiotic powder (10^10^ CFU), with 0.5%silicon dioxide added as a filler, and then vacuum-packaged. Five prototype samples were randomly selected and verified to contain 10^10^ CFU through viable cell counting. Subsequently, these products were stored at different temperatures (4, 15, 25, 35, and 45°C) for 24 wk, and viable cell counts were measured weekly using the aforementioned serial dilution method. The measured viable cell counts were expressed as log_10_ values and survival rates (%). Survivability was calculated using the formula: (%) survivability = [(initial viable cell count –after viable cell count) / initial viable cell count] × 100.

### Tolerance in a Simulated Gastrointestinal Tract

The acid and bile tolerance tests were performed as previously described, with minor modifications [8]. Briefly, each bacterial cell sample (~10^10^ CFU ml^-1^) was incubated at 37°C in a shaking water bath at 85 rpm in the following solutions: artificial saliva solution (30.0 g/l NaHCO_3_, 14.0 g/l KCl, 4.0 g/l CaCl_2_, and 2.0 g/l NaCl), simulated gastric solution (0.04 g pepsin in 0.1 mol/l HCl; pH 2 ± 0.2), and simulated intestinal solution (0.9 mol/l Na-bicarbonate, 0.04 g/ml bile salts glycodeoxycholate, 0.025 g/ml taurodeoxycholate, 0.04 g/ml taurocholate, and 0.08 g/l pancreatin; pH 7.5 ± 0.2) for designated time intervals. Subsequently, appropriate dilutions were plated directly onto MRS agar and incubated for 18 h at 37°C in 5% CO_2_ to determine the log10 CFU ml^-1^.

### Antioxidant Activity

The antioxidant activity of each probiotic and paraprobiotic was evaluated by measuring both the electron-donating ability and the cytoprotective capacity against reactive oxygen species (ROS) in Caco-2 cells. The 2,2-diphenyl-1-picrylhydrazyl (DPPH) radical scavenging assay was performed according to the method described by Rafiquzzaman *et al*. (2016) [16]. The hydroxyl radical (OH^•^) scavenging assay, superoxide anion radical (O_2_^•-^) scavenging assay, and Caco-2 cytoprotective activity were performed in accordance with the method described by Lee *et al*., (2020) [17]. Probiotics and paraprobiotics containing viable cells and biomass equivalent to 1010 /ml were used for the antioxidant activity assessments. Ascorbic acid (1 mg) served as a positive control. Optical density was measured using a microplate reader MRX A2000 (KLAB, Republic of Korea). The radical scavenging activity was calculated using the following formula: Radical scavenging activity (%) = [(Absorbance of the control−Absorbance of the sample)/Absorbance of the control] × 100.

The cytoprotective effect against ROS damage was determined by the coincubation method, with slight modification [17]. Briefly, Caco-2 cells were cultured in 48-well plates at a density of 1 × 10^6^ cells/well, and probiotics and paraprobiotics equivalent to 10^10^ CFU ml^-1^ bacterial cells were added to the culture wells. ROS generation was induced by adding 1 mM H_2_O_2_ to each well. In the control, H_2_O_2_ was replaced by PBS. After 3 h of incubation at 37°C in a 5% CO_2_ environment, cell viability was assessed via the MTT assay as described above.

### Nitric Oxide (NO) Production and Cytokine Assay for Assessing Anti-Inflammatory Effect

RAW 264.7 cells were seeded in a 96-well plate at a density of 2 × 10^5^ cells/well. Following an 18-h incubation period, the cells were treated with probiotics and paraprobiotics at a concentration equivalent to 10^10^ CFU ml^-1^, in the presence of 1 μg ml^-1^ lipopolysaccharides (LPS; Sigma-Aldrich), and incubated for an additional 24 h. The nitrite concentration in the cell supernatant, indicative of NO production, was measured using the Griess Reagent (1% sulfanilamide in 5% phosphoric acid and 0.1% naphthylethylenediamine dihydrochloride in water) method from the Nitrite Assay Kit (Sigma-Aldrich). Briefly, 100 μl of each supernatant was combined with 20 μl of Griess reagent and 80 μl of distilled water in a 96-well plate. The mixtures were incubated at 28°C for 1 h, after which the absorbance of each well was measured at 548 nm using a microplate reader. The nitrite concentration in the samples was calculated based on a sodium nitrite calibration curve (0–100 μM). As a positive control, 100 μM *N*ω-Nitro-L-arginine methyl ester hydrochloride (L-NAME), a known non-selective inhibitor of NO synthase, was used.

The measurement of pro-inflammatory cytokines, TNF-α and IL-6, was conducted as mentioned above by collecting cells following sample treatment, using the Mouse TNF-alpha ELISA Kit (ab208348; Abcam, UK) and the Mouse IL-6 ELISA Kit (ab222503; Abcam), in accordance with the manufacturer's protocol. A 10 μM concentration of dexamethasone (DEXA), a synthetic glucocorticoid that exerts anti-inflammatory effects, was used as a positive control.

### mRNA Expression Analysis

Gene expression levels were measured using real-time quantitative PCR (RT-qPCR) [12]. For the mRNA expression analysis of zonula occludens-1 (**ZO*-1*), claudin-1 (**CLDN*-1*), and occludin (*OCLN*) in Caco-2 cells, cells were suspended in fresh MEM without FBS and streptomycin/penicillin and seeded in 6-well tissue culture plates (Costar, Corning Inc., USA) at a density of 1 × 10^6^ cells/well. Once cells reached approximately 75%confluence, they were treated for 24 h with 100 μg/ml LPS, after which probiotics and paraprobiotics equivalent to 10^10^ CFU ml^-1^ bacterial cells were added to the cells with LPS for an additional 24 h. After the probiotic and paraprobiotic treatments, total RNA was isolated using the Riboclear Plus Kit (GeneAll Biotechnology, Republic of Korea). cDNA was synthesized from the isolated RNA template using the PrimeScript cDNA Synthesis Kit (TaKaRa Bio, Japan). Gene expression was quantified using TB Green Premix Ex Taq (TaKaRa Bio) on a TP700/ 760 Thermal Cycler Dice (TCD) Real-Time System (Takara). Relative expression levels were analyzed using the TCD software 5.0 with the 2^-ΔΔCT^ method, with glyceraldehyde 3-phosphate dehydrogenase (*GAPDH*) as the reference gene. The gene-specific primers used for amplification were as follows: **ZO*-1*, forward 5'-TTCACGCAGTTACGAGCAA-3' and reverse 5'-TTCACGCAGTTACGAGCAA-3'; **CLDN*-1*, forward 5'-TGGTCAGGCTCTCTTCACTG-3' and reverse 5'-TTGGATAGGGCCTTGGTGTT-3'; *OCLN*, forward 5'-TTGGATAGGGCCTTGGTGTT-3' and reverse 5'-GCCTGTAAGGAGGTGGACT-3'; *GAPDH*, forward 5'-GATGCTGGCGCTGAGTA-3' and reverse 5'-GGCAGAGATGATGACCCT-3'. Additionally, after the animal experiments, the gene expressions of *Zo*-1, *mucin*-1 (*Muc*-1), *Tnf*-α, and *Il*-6 were analyzed from the colonic tissues of each group. The total RNA extraction, cDNA synthesis, and reference genes are the same as mentioned above, and the gene-specific primers were as follows : *Zo*-1, forward 5'-AGGTACAGGCCAGAGGCACA-3' and reverse 5'-CAGCACCATGGAGAGGCTCG-3'; *Muc*-1, forward 5'-GTCAATACCCTGTTTCTCCTACC-3' and reverse 5'-GGGTGAACTGTTACTGGAGAAG-3'; *Tnf*-α, forward 5'-AAGAGGCACTCCCCCAAAAG-3' and reverse 5'-ATCCCTTTGGGGACCGATCA-3'; *Il*-6, forward 5'-CATCCAAACATCCTCCCCCAA-3' and reverse 5'-AGGCGGCTTAGTTAGATCCCT-3'; *Gapdh*, forward 5'-GAAGGTCGGTGTGAACGGAT-3' and reverse 5'-ACTGTGCCGTTGAATTTGCC-3'.

### Animals, Diets, and Experimental Design

**Fig. 1:**
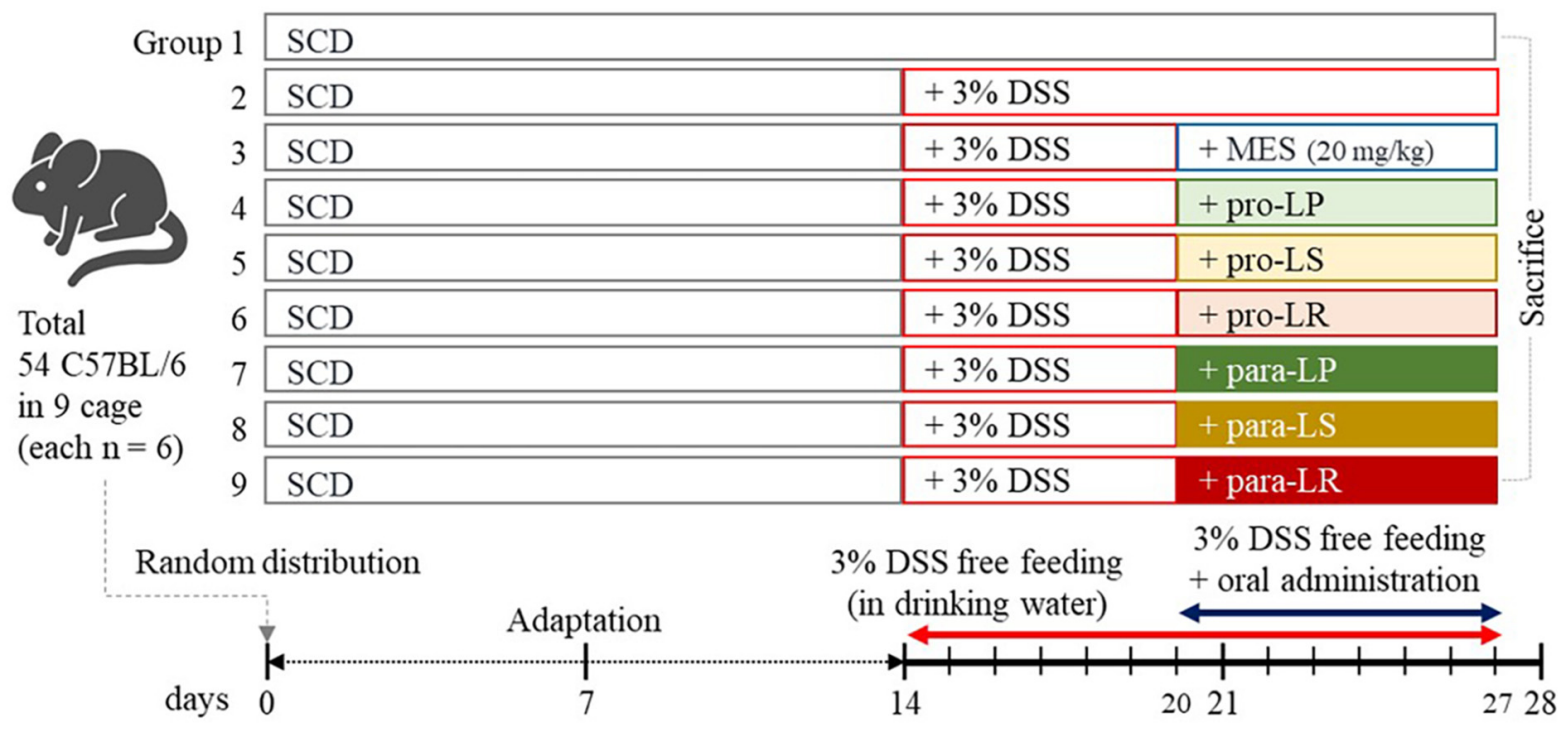
Schematic diagram illustrating the animal grouping and experimental procedures used for the comparative analysis of the protective effects of probiotics and paraprobiotics in DSS-induced colitis.

Six-week-old specific pathogen-free (SPF) grade C57BL/6 male mice (*n* = 54, 21.4 ± 2.3 g) were obtained from Samtako Bio Korea Co., Ltd. (Republic of Korea). The mice were housed at 22 ± 2°C and 55 ± 5% humidity under a 12-h light/dark cycle. After a 2-wk adaptation period, the mice were assigned to nine groups (*n* = 6 per group):control (standard chow diet; SCD), negative control (dextran sulfate sodium; DSS), positive control (mesalazine; 5-aminosalicylic acid; MES), and three groups each for probiotics and paraprobiotics (*L. plantarum* MGEL20154; pro-LP and para-LP, *L. sakei* MGEL23040; pro-LS and para-LS, and *L. reuteri* MGEL21001; pro-LR and para-LR). Control groups received SCD alone (10% calories from fat, Diet D12450J, Research Diets Inc., USA), SCD with 3%DSS (free feeding in drinking water), or SCD with 3% DSS and 20 mg/kg MES. Experimental groups received viable and heat-killed cells corresponding to 10^10^ CFU in 200 μl/mouse/day for each strain via a gastric tube orally. The experimental design is summarized in Figure 1.

### Histological Analysis and Myeloperoxidase (MPO) Assay

Colon tissues were sampled from each group, fixed in 4% paraformaldehyde (PFA), and embedded in paraffin. Then, 5-μm-thick sections were prepared and stained with hematoxylin and eosin (H&E). Histological features were observed using a Leica microscope (DM2500 LED; Leica, Germany). Histological scores were calculated according to the method described by Erben *et al*., 2014 [18]. The degree of inflammation in the colon was compared based on the sum of the immune cell infiltration score (0–3 points) and the morphological damage score of the intestinal mucosa (0–3 points), according to the criteria outlined in Table 1. For MPO assay, colon tissues were homogenized in 0.5% cetyltrimethylammonium bromide (CTAB), then centrifuged at 15,000 ×*g* at 4°C for 20 min for MPO assay. A 0.1 ml aliquot of supernatant was mixed with 2.9 ml of PBS containing 0.0005%hydrogen peroxide and 0.167mg/ml O-dianisidine dihydrochloride. MPO activity was calculated from optical density changes at 460 nm at 25°C, with one unit of MPO defined as the amount that degrades 1 μ/mol of H_2_O_2_ per minute. activity was expressed as units/mg of protein.

### Statistical Analysis

All data were analyzed by one-way analysis of variance (ANOVA) using the Statistical Package for the Social Sciences (SPSS) followed by Duncan’s multiple range test. Statistical significance was determined at a *p*-value < 0.05 unless otherwise noted.

## Results

### Bacterial Identification

**Fig. 2:**
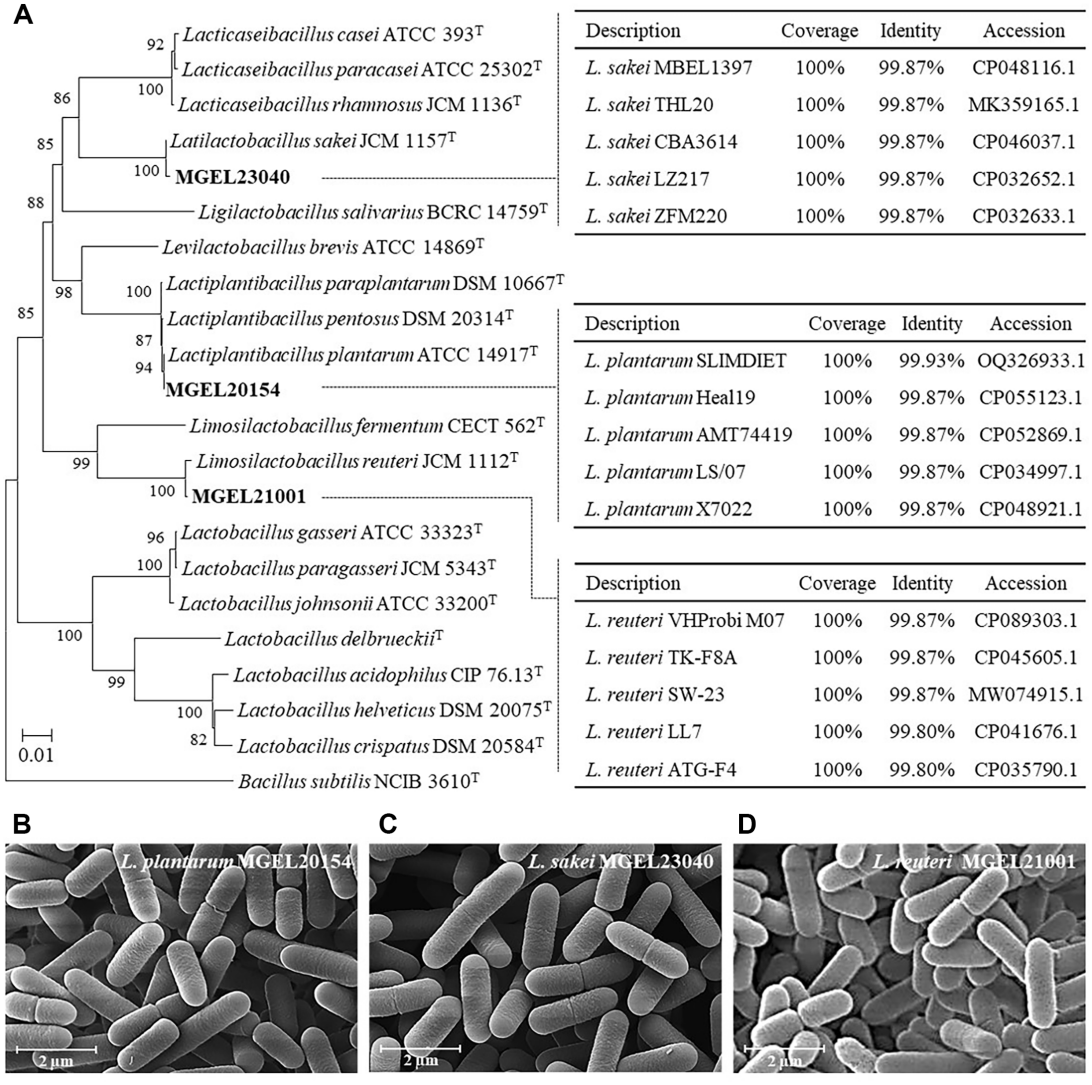
Phylogenetic taxonomy and morphological characteristics of each strain. (**A**) Phylogenetic tree based on 16S rRNA sequences constructed using the neighbor-joining method, showing the position of the strains relative to type species of representative lactic acid bacteria. Bootstrap percentages (based on 1,000 replicates) are indicated at the nodes. Scale bar: 5 substitutions per 1,000 nucleotide positions. (**B–D**) Scanning electron micrographs of MGEL20154, MGEL23040, and MGEL21001, respectively.

Phylogenetic analysis of the 16S rRNA gene sequences (>1505 bp) from representative type strains confirmed that the strains MGEL20154, MGEL23040, and MGEL21001 belong to the genera *Lactiplantibacillus*, *Latilactobacillus*, and *Limosilactobacillus*, respectively. The strains showed the highest 16S rRNA gene sequence similarity to *L. plantarum* SLIMDIET (99.93%; accession no. OQ326933.1), *L. sakei* MBEL1397 (99.87%; CP048116.1), and *L. reuteri* VHProbi M07 (99.87%; CP089303.1) (Fig. 2). Morphologically, the three strains exhibited typical characteristics of lactic acid bacteria, appearing as opaque-creamy yellow, rod-shaped cells with sizes ranging from 1.0 to 1.8 μm on MRS agar media.

### Resistance to Various Temperatures and Simulated Gastrointestinal Conditions

**Fig. 3:**
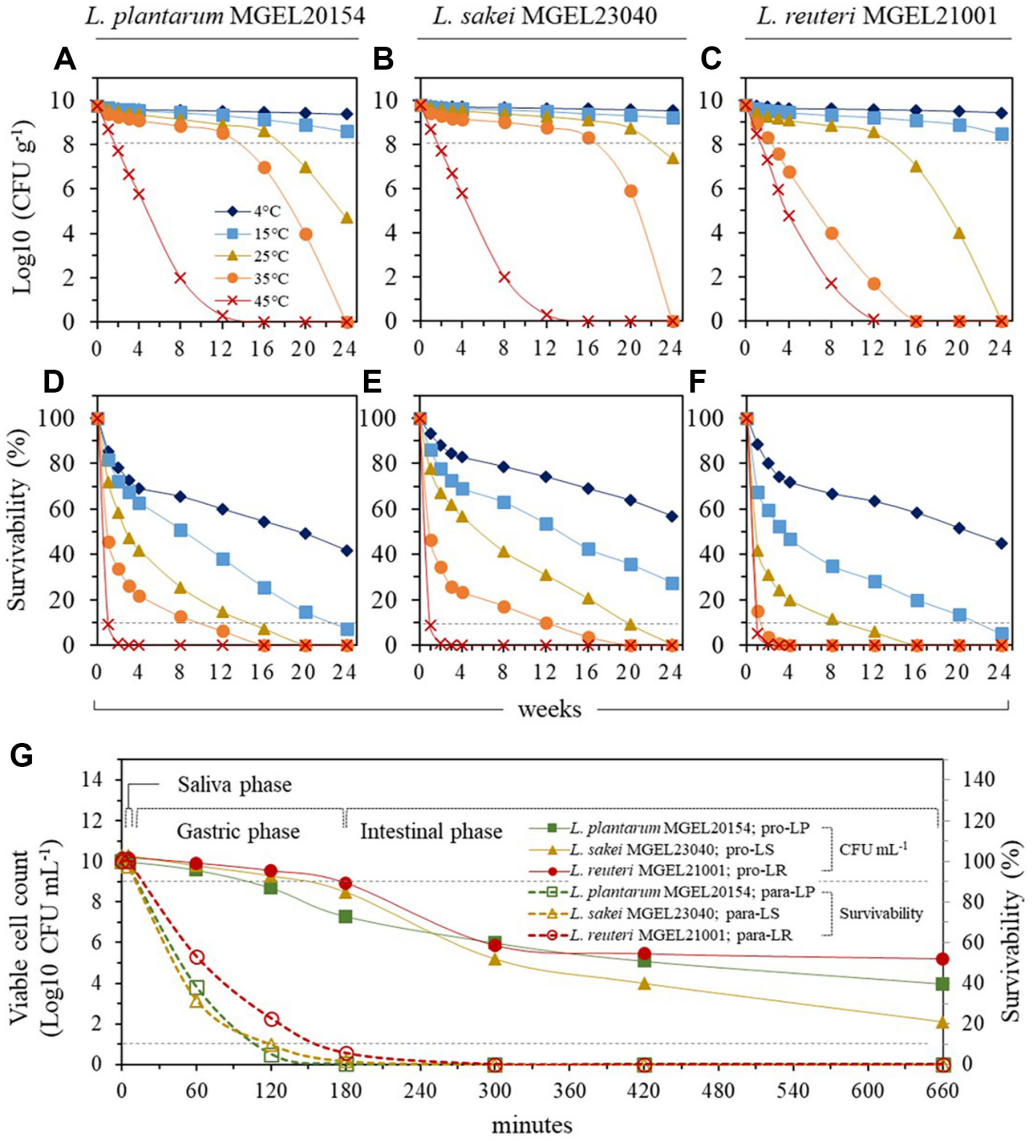
Stability of strains at various temperatures and under simulated gastrointestinal conditions. (**A–C**) Log10 CFU g^-1^ values and (**D–F**) survival rates of MGEL20154, MGEL23040, and MGEL21001 over 24 wk at 4°C, 15°C, 25°C, 35°C, and 45°C. (**G**) Survival rates of MGEL20154, MGEL23040, and MGEL21001 over time under simulated gastrointestinal tract conditions. Standard deviations for these values are less than 3%.

The stability of probiotic strains was evaluated over 24 weeks at various temperatures and for 11 h in a simulated gastrointestinal environment (Fig. 3). The survival rates of all three strains decreased progressively from the first wk at each tested temperature. After 24 wk, no viable cells were detected for any strain at 35°C and 45°C (Fig. 3A-3C). At temperatures above 15°C, MGEL20154 and MGEL21001 exhibited survival rates below 10% after 24 wk, whereas MGEL23040 showed a survival rate of 27.6% (Fig. 3D-3F). At 4°C, the survival rates after 24 wk were 24.4% for MGEL20154, 56.9% for MGEL23040, and 45.0% for MGEL21001. In the simulated gastrointestinal tract environment, all strains showed a survival rate over 99.9% after the saliva phase. However, during the gastric phase, survival rates declined sharply, resulting in less than 10% survival for all three strains post-gastric phase (Fig. 3G). During the intestinal phase, the survival rates of all strains gradually decreased over time. After 11 h in the simulated gastrointestinal tract, the final viable cell counts were 10^4^ for MGEL20154, 10^2^ for MGEL23040, and 10^5^ for MGEL21001, reflecting a survival rate of less than 10^4^ compared to the initial viable cell count of 10^10^ CFU ml^-1^.

### Antioxidant Effects of Probiotics and Paraprobiotics

**Fig. 4:**
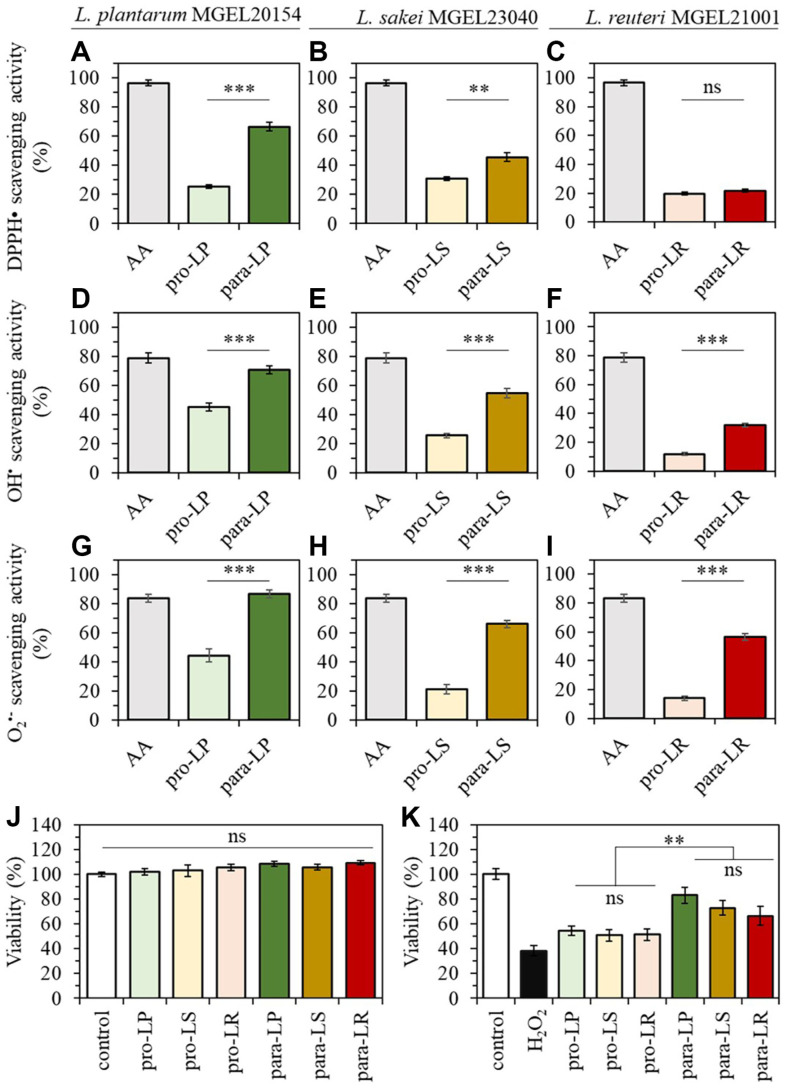
Antioxidant effects of probiotics and paraprobiotics. (**A–C**) DPPH radical scavenging, (**D–F**) OH^•^ scavenging, and (**G–I**) O_2_^•-^ scavenging activity for MGEL20154, MGEL23040, and MGEL21001. (**J**) Cytotoxicity effects of each probiotic and paraprobiotic on Caco-2 cells. (**K**) Cytoprotective effects of probiotics and para probiotics on Caco-2 cells under hydrogen peroxide-induced oxidative stress. In these panels, pro-LP, pro-LS, and pro-LR refer to the viable cells of MGEL20154, MGEL23040, and MGEL21001, respectively, while para-LP, para-LS, and para-LR refer to the heat-killed cells of MGEL20154, MGEL23040, and MGEL21001, respectively. *p < 0.05, **p < 0.01 and ***p < 0.001 versus the other group. The data represent mean ± SEM. *n* = 5. ns: not significant, as indicated.

The antioxidant activities of probiotics and paraprobiotics, corresponding to 10^10^ CFU ml^-1^, are shown in Fig. 4. Except for the DPPH radical scavenging activity of MGEL21001, all antioxidant assays demonstrated significantly higher activity for paraprobiotics compared to probiotics. Specifically, the paraprobiotics of MGEL20154 exhibited DPPH radical, OH^•^, and O_2_^•-^ scavenging activities corresponding to 71.1%, 95.9%, and 103.6% of 1 mg ascorbic acid, respectively, demonstrating the highest antioxidant capacity among the three strains (Fig. 4A-4I). Furthermore, the antioxidant capacity of all paraprobiotics was, on average, 2.3 times higher than that of probiotics. The cytotoxicity and protective effects of probiotics and paraprobiotics against ROS on Caco-2 cells are illustrated in Figure 4J and 4K. The *in vitro* cytotoxicity analysis using the MTT assay revealed that Caco-2 cell viability exhibited no growth inhibition or cytotoxic effects for any of the tested probiotics and paraprobiotics. Following exposure to 1 mM H_2_O_2_ for 3 h, the viability of Caco-2 cells significantly decreased to approximately 38.34 ± 4.02% compared to the untreated group. In contrast, pre-treatment with each probiotic and paraprobiotic demonstrated protective effects on Caco-2 cells, with cell protection rates of 54.7 ± 3.8%, 50.8 ± 4.7%, and 51.4 ± 4.6% for the probiotics (pro-LP, pro-LS, and pro-LR), and 83.0 ± 6.2%, 72.9 ± 5.8%, and 66.5 ± 7.7% for the paraprobiotics (para-LP, para-LS, and para-LR), respectively. Therefore, paraprobiotics exhibited an average cytoprotective effect approximately 1.4 times greater than that of probiotics.

### Anti-Inflammatory Effects in LPS-Stimulated RAW 264.7 Macrophages

**Fig. 5:**
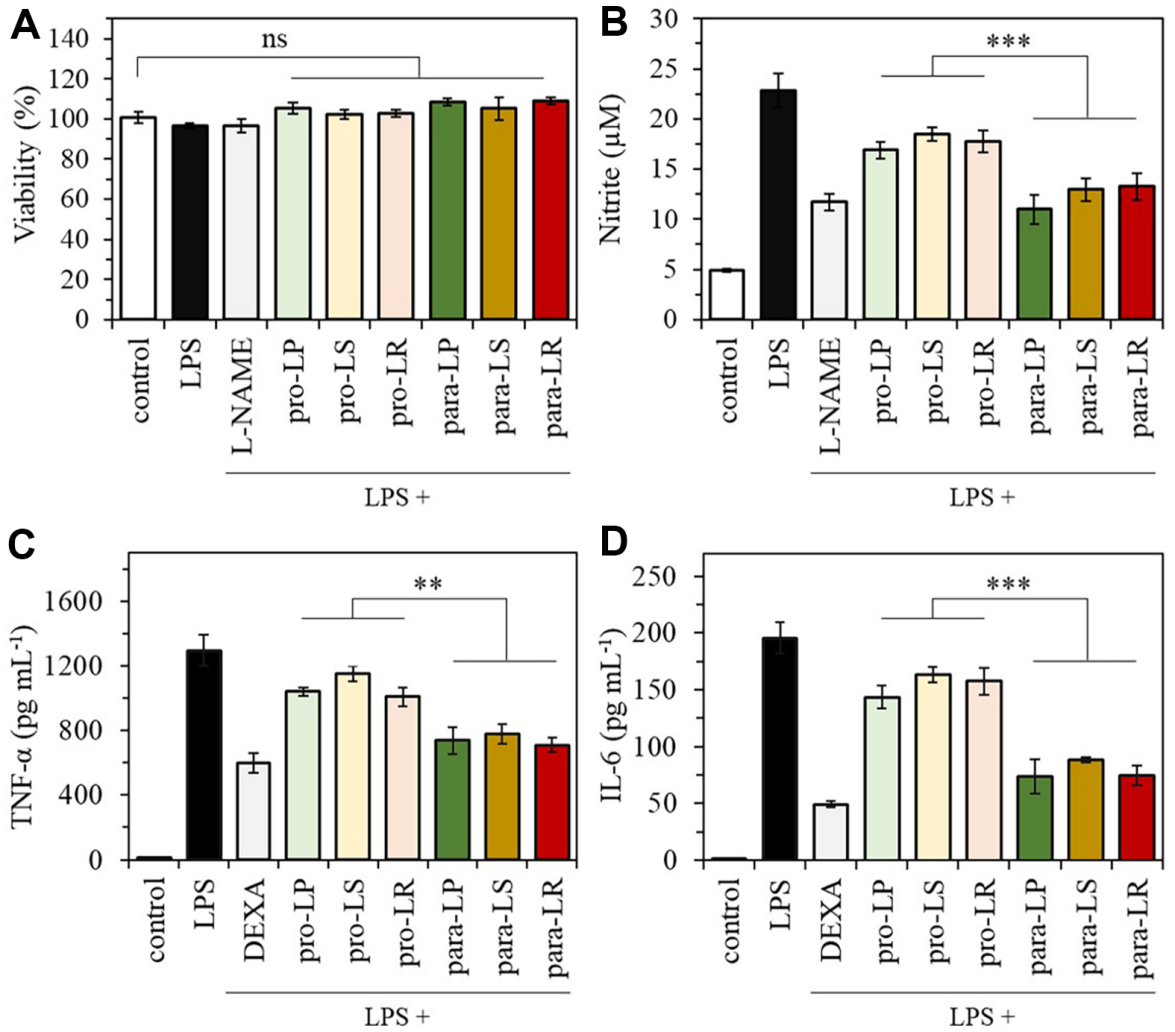
Anti-inflammatory effects in LPS-stimulated RAW 264.7 macrophages. (**A**) Cytotoxicity of each probiotic and paraprobiotic against RAW 264.7 cells, (**B**) NO production and the inhibitory effects on pro-inflammatory cytokines (**C**) TNF-α and (**D**) IL-6 in LPS-stimulated RAW 264.7 cells. *p < 0.05, **p < 0.01 and ***p < 0.001 versus the other group. The data represent mean ± SEM. *n* = 5. ns: not significant, as indicated.

No cytotoxicity was observed for either the probiotics or paraprobiotics at a concentration of 10^10^ CFU ml^-1^ against RAW 264.7 mouse macrophage cells (Fig. 5A). Both probiotics and paraprobiotics groups significantly inhibited NO production. Although no significant difference was observed within each probiotic or paraprobiotic group, a comparison between the two groups indicated that the paraprobiotics group (para-LP: 11.01 ± 1.45 μM, para-LS: 12.96 ± 1.12 μM, and para-LR: 13.26 ± 1.38 μM) demonstrated a substantial inhibition, comparable to that of the 100 μM L-NAME (11.76 ± 0.84 μM) (Fig. 5B). The levels of pro-inflammatory cytokines TNF-α and IL-6 were more rapidly inhibited in the paraprobiotics group than in the probiotics group. In particular, TNF-α inhibition by paraprobiotics was comparable to that achieved with DEXA (600.51 ± 59.37 pg ml^-1^), with para-LP at 736.70 ± 82.09 pg ml^-1^, para-LS at 777.56 ± 59.66 pg ml^-1^, and para-LR at 710.59 ± 46.69 pg ml^-1^ (Fig. 5C). Additionally, the paraprobiotics group showed substantial IL-6 inhibition (para-LP; 73.68 ± 15.12 pg ml^-1^, para-LS; 88.40 ± 2.17 pg ml^-1^, and para-LR; 74.41 ± 8.95 pg ml^-1^), achieving reductions exceeding 180% compared to the probiotics group (pro-LP; 143.48 ± 9.99 pg ml^-1^, pro-LS; 163.24 ± 6.60 pg ml^-1^, and pro-LR; 157.32 ± 12.21 pg ml^-1^)(Fig. 5D). These results indicate that the paraprobiotics from the three tested strains demonstrated a more potent anti-inflammatory effect, effectively inhibiting NO and cytokine production in LPS-activated RAW 264.7 macrophages compared to their probiotic counterparts.

### Recovery of Tight Junctions in LPS-Induced Damage in Caco-2 Cells

**Fig. 6:**
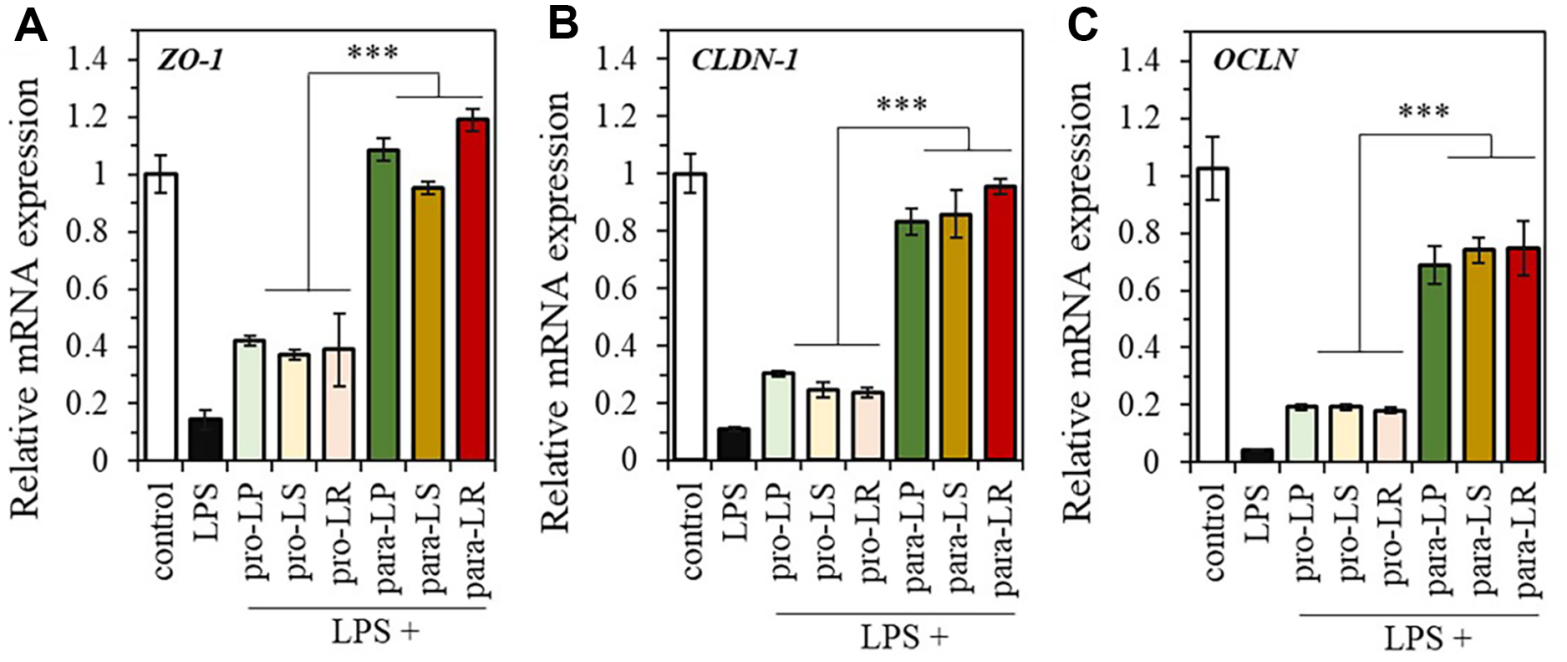
Recovery of tight junctions in LPS-induced damage in Caco-2 cells. mRNA expression of (**A**) *ZO*-1, (**B**) *CLDN*-1, and (**C**) *OCLN* in LPS-induced Caco-2 cells treated with each probiotic and paraprobiotic. *p < 0.05, **p < 0.01 and ***p < 0.001 versus the other group. The data represent mean ± SEM. *n* = 5. ns: not significant, as indicated.

LPS-induced damage in Caco-2 cells was clearly demonstrated by a decrease in the expression of the *ZO*-1, *CLDN*-1, and *OCLN* genes, which are involved in epithelial barrier integrity (Fig. 6). The mRNA expression of *ZO*-1, *CLDN*-1, and *OCLN* recovered more rapidly in the paraprobiotic treatment group compared to the probiotic group. Specifically, the key tight junction protein, *ZO*-1, exhibited mRNA expression levels in the paraprobiotic group (para-LP: 1.09 ± 0.04, para-LS: 0.95 ± 0.02, and para-LR: 1.19 ± 0.04) that were significantly similar to those of normal cells not treated with LPS (Fig. 6A). Although there were no significant differences in mRNA expression levels of *ZO*-1, *CLDN*-1, and *OCLN* within each probiotic or paraprobiotic group, the expression differences between the probiotic and paraprobiotic groups revealed that *ZO*-1 was upregulated by 2.73-fold, *CLDN*-1 by 3.42-fold, and *OCLN* by 3.81-fold in the paraprobiotic group (Fig. 6B and 6C). Therefore, the tested paraprobiotics effectively upregulated gene expression that had been reduced due to LPS-induced disruption of intestinal integrity, in comparison to the probiotics.

### Mitigation of Colonic Tissue Damage and Colonic MPO Activity in DSS-Induced Colitis Mice

**Fig. 7:**
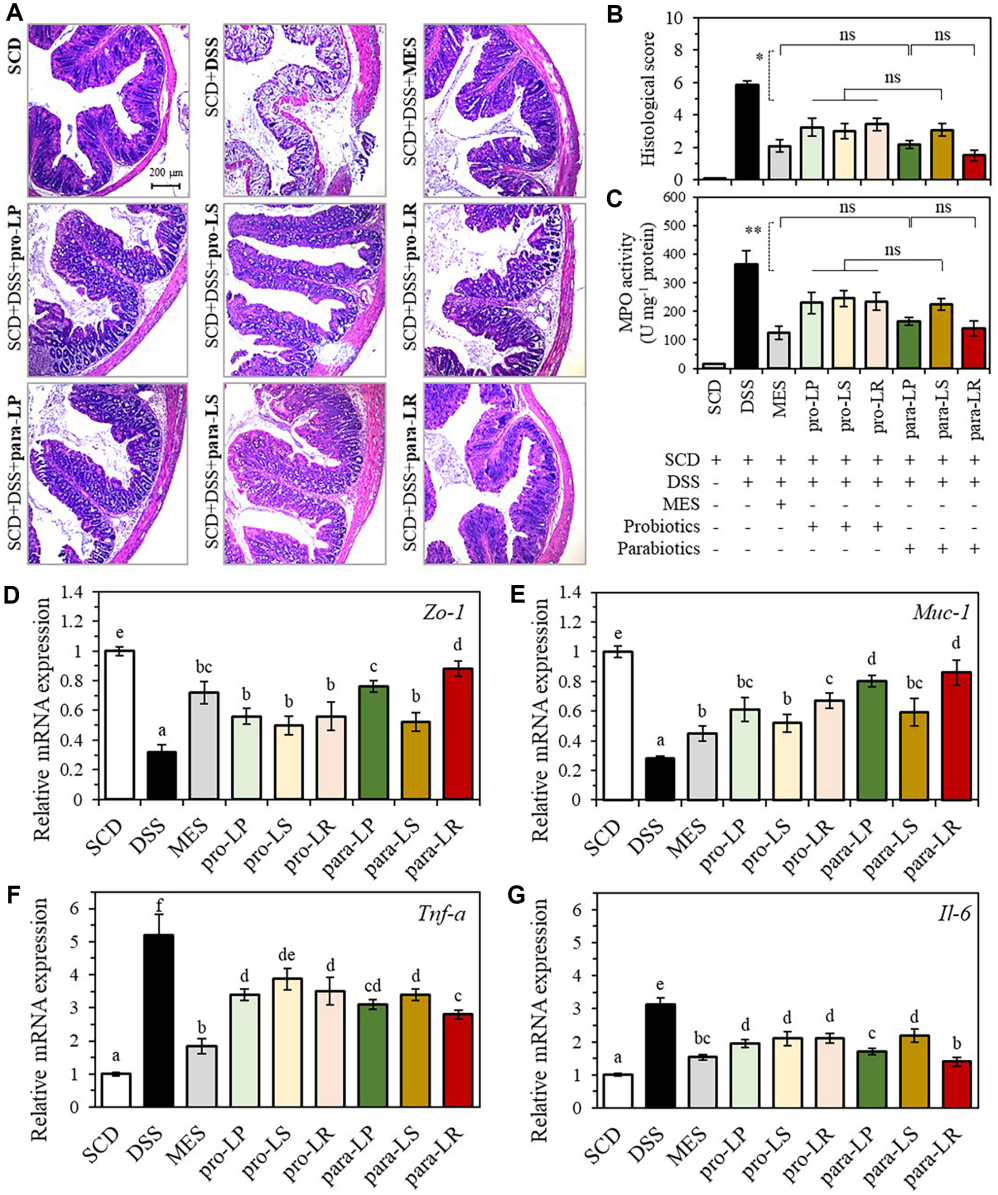
Comparison of the ameliorative effects of probiotics and paraprobiotics on histopathological changes and myeloperoxidase (MPO) activity in the colons of mice with DSS-induced colitis. (**A**) Representative images of H&E-stained colonic tissues from each group. (**B**) Histological scores. (**C**) MPO activity. (**D-G**) Relative mRNA expression of *Zo*-1, *Muc*-1, *Tnf*-α, and *Il*-6 in the colon tissues. *p < 0.05, **p < 0.01 and ***p < 0.001 versus the other group. The data represent mean ± SEM. *n* = 6. ns: not significant, as indicated.

As shown in Fig. 7A, the group administered only 3.0% DSS exhibited extensive colonic tissue damage, including immune cell infiltration, lesion formation, villous destruction, and increased colonic muscle thickness. In contrast, the colon tissue of the group fed only SCD appeared normal. Treatment with MES, a drug widely used for inflammatory bowel disease, significantly villi structure and alleviated inflammatory responses such as mucosal and submucosal infiltration. Furthermore, the group that consumed probiotics and paraprobiotics equivalent to 10^10^ CFU for one wk showed significant alleviation of all signs of colonic tissue damage, including immune cell infiltration, lesion formation, villous destruction, and increased colonic muscle thickness. Histological scores derived from observations of inflammatory cell infiltration and intestinal architecture in the colonic tissues of 54 mice across nine groups were significantly lower in all groups compared to the DSS group (5.8 ± 0.3), particularly in the para-LP (2.2 ± 0.3) and para-LR (1.5 ± 0.3) groups, which recorded histological scores similar to MES (2.1 ± 0.4) (Fig. 7B). With the progression of colonic inflammation, the MPO levels in the DSS group were 365.24 ± 48.15 U mg^-1^, whereas the MES treatment group showed a decrease to 124.82 ± 23.65 U mg^-1^ (Fig. 7C). Similar to the histological scores, the pro-LP, pro-LS, pro-LR, and para-LS groups showed significant reductions compared to the DSS group, with values of 231.39 ± 36.21 U mg^-1^, 244 ± 27.84 U mg^-1^, 234.83 ± 31.30 U mg^-1^, and 225.33 ± 19.97 U mg^-1^, respectively. In contrast, the para-LP (165.67 ± 14.78 U mg^-1^) and para-LR (140.17 ± 27.66 U mg^-1^) groups exhibited MPO activities similar to those of MES. Thus, among the tested groups, the intake of para-LP and para-LR in DSS-induced colitis mice most effectively suppressed colonic immune cell infiltration.

### Improvement of Intestinal Barrier Function and Anti-Inflammatory Effects in DSS-Induced Colitis Mice

The mRNA expression levels in the colonic lesion tissues of each group were analyzed to investigate the improvement of intestinal function and the anti-inflammatory effects of each type of biotic. The relative mRNA expression level of *Zo*-1 was downregulated to 0.32 ± 0.04 in the DSS group, while it was restored to 0.72 ± 0.07 in the MES group (Fig. 7D). In the pro-LP, pro-LS, and pro-LR groups, the expression levels were significantly upregulated to 0.56 ± 0.05, 0.51 ± 0.06, and 0.52 ± 0.09, respectively, compared to the DSS group. However, no significant differences were observed among the probiotic groups. The para-LP and para-LR groups demonstrated significant upregulation, with levels of 0.76 ± 0.03 and 0.88 ± 0.05, respectively, while the para-LS group exhibited *Zo*-1 expression levels similar to those of the probiotic group. The relative mRNA expression level of *Muc*-1, a mucin protein found in the epithelial cells of the intestinal mucosa, was drastically downregulated to 0.28 ± 0.01 in the DSS group and significantly upregulated to 0.45 ± 0.05 in the MES group (Fig. 7E). In particular, among the probiotics and paraprobiotics groups, the para-LP and para-LR groups exhibited significantly upregulated expression levels of 0.81 ± 0.04 and 0.86 ± 0.08, respectively, which were closer to the levels observed in the normal (SCD) group. The expression of inflammation markers *Tnf*-α and *Il*-6 in the colonic lesion tissues were presented in Figure 7F and 7G. Both *Tnf*-α and *Il*-6 were upregulated to 5.21 ± 0.62 and 3.12 ± 0.21, respectively, in the DSS group, and significantly downregulated to 1.84 ± 0.24 and 1.54 ± 0.07 in the MES group. The biotic groups exhibited similar expression patterns for *Tnf*-α and il-6, with the para-LP and para-LR groups demonstrating the most effective downregulation. Meanwhile, no significant differences were observed in the expression levels of *Tnf*-α and *Il*-6 between the para-LR group and the probiotic groups. In conclusion, consistent with the results of the histopathological analysis and MPO assay, the para-LP and para-LR groups were found to be the most effective in restoring tight junctions and mucin secretion, as well as in reducing inflammation in colonic lesion tissues. Furthermore, this suggests that the *in vitro* results demonstrating that paraprobiotics exhibit similar or superior antioxidant, anti-inflammatory, and tight junction-enhancing efficacy compared to probiotics have been validated by *in vivo* evidence.

## Discussion

The three bacterial strains used in this study were isolated in our laboratory and verified for their effectiveness in alleviating metabolic diseases and regulating the gut microbiome. However, we have recognized that frequent cultivation is required to maintain freshness and survival rates of live bacteria, and this, along with limitations caused by genetic mutations due to frequent subculturing, presents significant economic disadvantages for industrial applications. The limitation survival persistence in live bacteria is evident in the stability tests conducted in this study, which examined the temperature and pH sensitivity of these probiotics. Although only the stability test results for the three strains used in this study are presented here, extensive stability tests conducted by our research team on over 500 strains over an extended period indicate that nearly all tested probiotics survived at room temperature (18–25°C) for a maximum of six months. Additionally, in environments with a pH below 2, although inter-strain differences emerged after 3 h of exposure, the average survival rate remained below 0.0001%. Previous studies report that the survival rate of probiotics is up to one year at 4–10°C, approximately 3–6 months at 20–25°C, and 1–3 months at 30–40°C [19, 20]. These findings clearly support our conclusion that probiotics stored at temperatures above 15°C for over six months are unlikely to retain any beneficial effects upon consumption. Generally, lactic acid bacteria, widely used as probiotics for human consumption, are considered acid-resistant; however, their sensitivity significantly increases below pH 3 [21]. Moreover, in a highly acidic environment below pH 2, particularly in the presence of gastric secretions (such as pepsin and trypsin), it is unlikely that these bacteria can maintain a survival rate sufficient to confer health benefits [8]. Furthermore, one of our primary concerns regarding live bacterial products is the risk of unauthorized release of functional live bacteria to gain a competitive advantage in the rapidly expanding global market for probiotics and LBPs, despite being protected by intellectual property rights. This risk threatens the motivation to secure and develop novel, superior strains. Therefore, to address these limitations associated with live bacteria, we conducted this study to quantitatively compare and analyze the functions of functional probiotics, with partially proven efficacy in animal models, alongside the dead-cell paraprobiotics.

In the *in vitro* evaluation of functional aspects of the tested probiotics and paraprobiotics, paraprobiotics from the three strains demonstrated effects equal to or superior to those of probiotics in terms of antioxidant activity, anti-inflammatory effects, and enhancement of tight junction integrity. All tested paraprobiotics exhibited significantly higher radical scavenging activity and protective effects against ROS-induced damage in intestinal cells compared to probiotics. Notably, the MGEL20154 strain (10^10^ CFU) showed antioxidant activity comparable to that of ascorbic acid. The study further confirmed that paraprobiotics possess more effective anti-inflammatory capabilities and greater efficacy in promoting tight junction recovery in macrophages and epithelial cells challenged with endotoxins. Furthermore, consistent with *in vitro* findings, both probiotics and paraprobiotics were effective in improving histological scores and reducing MPO levels in a DSS-induced ulcerative colitis mouse model. Notably, paraprobiotics derived from MGEL20154 and MGEL21001 demonstrated significantly enhanced efficacy in restoring tight junctions, promoting mucin secretion, and reducing inflammation in colonic lesion tissues compared to their probiotic counterparts. These findings suggest that paraprobiotics may be more effective in alleviating ulcerative colitis *in vivo*, demonstrating functional roles in antioxidant activity, anti-inflammatory effects, and the enhancement of intestinal barrier integrity.

A plausible hypothesis for the significantly higher antioxidant, anti-inflammatory, and intestinal integrity-recovery effects observed in paraprobiotics compared to probiotics is that active compounds, such as phospholipids, fatty acids, and oligosaccharides, contained in the internal structures and cell membranes of non-viable bacterial cells are released and exist in an activated state. For instance, the immunomodulatory function of *Akkermansia muciniphila* in the host has been shown to be associated with diacyl phosphatidylethanolamine (a15:0-i15:0 PE) in its bacterial cell membrane [22]. This phospholipid may modulate immune response by lowering the activation threshold for immune signaling through the induction of low levels of inflammatory cytokines, such as TNFα and IL-6, indicating its potential for immune modulation. Additionally, β-glucan/galactan (CSGG) derived from *Bifidobacterium bifidum* has been identified as a key component in the induction of Foxp3+ regulatory T cells (Treg cells) [23]. CSGG enhances the production of inhibitory cytokines by promoting Treg cell induction through a dendritic cell-dependent mechanism. Detection of CSGG by Toll-like receptor 2 (TLR2) on dendritic cells is critical for inducing Treg cell induction, suggesting that CSGG can suppress intestinal inflammation and may serve as a therapeutic agent for immune homeostasis and allergic diseases. Furthermore, peptidoglycan and muropeptides derived from *Lactobacillus salivarius* are key substances for alleviating inflammatory bowel disease and have been shown to induce IL-10-producing dendritic cells that upregulate the immunosuppressive pathway of indoleamine 2,3-dioxygenase [24]. These findings strongly support our observation that the physiological effects of paraprobiotics, attributed to the release of phospholipids, oligosaccharides, and peptidoglycan-like compounds from non-viable cells, exhibit significant antioxidant, anti-inflammatory, and intestinal integrity-recovery activities. They also reinforce the hypothesis that non-viable cells can overcome the limitations of live probiotics while exhibiting superior physiological functionality.

Based on the results of this study, we assert that specific paraprobiotics can surpass the limitations of probiotics while demonstrating equal or superior efficacy in the alleviation and treatment of ulcerative colitis. Current therapeutic approaches for ulcerative colitis, a form of refractory inflammatory bowel disease, primarily focus on inflammation management and symptom alleviation. Treatments such as 5-aminosalicylic acid (5-ASA), corticosteroids, and immunomodulators are commonly used; however, these methods carry significant risks of side effects and recurrent symptom exacerbation [25]. Consequently, probiotic prescriptions are gaining attention. By targeting one of the underlying causes of ulcerative colitis—gut microbiome dysbiosis-probiotics are increasingly recognized not only for symptom management but also for addressing possible root causes of the disease [26, 27]. While the clinical efficacy of probiotics for patients with ulcerative colitis has been significantly validated [28-31], notable potential side effects cannot be disregarded [32, 33]. Specifically, for patients with ulcerative colitis, whose intestinal barrier is already severely compromised, live probiotic bacteria may cross the intestinal epithelium and enter the bloodstream, potentially triggering a systemic inflammatory response known as bacteremia. Additionally, excessive production of organic acids may exacerbate existing inflammation. In contrast, paraprobiotics allow for precise dosage adjustments that align with the host’s physiological needs, offering the potential to restore gut microbial balance, exert anti-inflammatory effects, and enhance barrier function, thereby inhibiting disease progression and alleviating symptoms. However, clear limitations exist in our research. Further studies involving a greater variety of strains and concentrations are required, and even probiotics that are considered safe at generally recognized as safe (GRAS) levels must be verified for their stability as paraprobiotics. Building on this foundational investigation, future research will focus on elucidating the specific mechanisms of action of paraprobiotics regarding their antioxidant, anti-inflammatory, and barrier-protective effects, as well as identifying the specific components responsible for these effects. Additionally, it is essential to investigate the impact of paraprobiotics on gut microbial communities through metagenomic analysis, bringing us one step closer to developing therapeutic agents aimed at curing refractory ulcerative colitis.

## Figures and Tables

**Table 1:** Histological scores for colitis.

Inflammatory cell infiltration		Score A
Severity	Extent	
Mild	Mucosa	1
Moderate	Mucosa & submucosa	2
Marked	Transmural	3
Intestinal architecture		Score B
Epithelial changes	Mucosal architecture	
Focal erosion		1
Erosion	± Focal ulcerations	2
	Extended ulceration	3
	± granulation tissue	
	± pseudopolyps	
	± Focal ulcerations	
Histological score	Score A + Score B	0 - 6
